# An Insight into Vaginal Microbiome Techniques

**DOI:** 10.3390/life11111229

**Published:** 2021-11-13

**Authors:** Mahima Sharma, Chitrakshi Chopra, Malvika Mehta, Varun Sharma, Sharada Mallubhotla, Srinivas Sistla, Jyothi C. Sistla, Indu Bhushan

**Affiliations:** 1 School of Biotechnology, Shri Mata Vaishno Devi University, Katra 182320, India; mahisharma2403@gmail.com (M.S.); chitrakshi288@gmail.com (C.C.); malvikamehta9@gmail.com (M.M.); sharda.p@smvdu.ac.in (S.M.); 2 Ancient DNA Laboratory, Birbal Sahni Institute of Palaeosciences, Lucknow 226007, India; sharmavarun840@gmail.com; 3 Department of Microbiology and Immunology, Stony Brook University, New York, NY 11790, USA; srinivas.sistla@stonybrook.edu; 4 Institute of Structural Biology, Drug Discovery and Development, Virginia Commonwealth University, Richmond, VA 23219, USA; jsistla@vcu.edu

**Keywords:** microbiome, preterm birth, bacterial vaginosis, diagnostic techniques, *Lactobacilli*, fungus, infertility

## Abstract

There is a unique microbial community in the female lower genital tract known as the vaginal microbiota, which varies in composition and density and provides significant benefits during pregnancy, reproductive cyclicity, healthy newborn delivery, protection from preterm birth, infections such as UTIs, bacterial vaginosis, and so on, and improves the efficacy of treatments for vaginal cancers. **Methods:** It is necessary to know how the vaginal microbiome is composed in order to make an accurate diagnosis of the diseases listed above. A microbiome’s members are difficult to classify, and the way microbial communities function and influence host–pathogen interactions are difficult to understand. More and more metagenomic studies are able to unravel such complexities due to advances in high-throughput sequencing and bioinformatics. When it comes to vaginal microbiota research, we’ll be looking at the use of modern techniques and strategies that can be used to investigate variations in vaginal microbiota in order to detect diseases earlier, better treat vaginal disorders, and boost women’s health. **Discussion:** The discussed techniques and strategies may improve the treatment of vaginal disorders and may be beneficial for women’s overall health.

## 1. Introduction

The human microbiome has gotten a lot of attention in the last decade, with studies beginning to confirm its role in human health. The microbiome contains trillions of microbial entities [1]. The communication of human microbiota with the environment is an interesting study as it provides a platform for understanding the impact of a specific host on the milieu. A detailed grasp of the normal vaginal microbiome is necessary for comprehending and identifying microbiome alterations linked with aberrant lower genital tract symptoms. The vaginal microbiota, which varies in composition and density, is found in the genital tract and promotes healthy pregnancy, reproductive cyclicity, and healthy births [2]. Increased lactic acid-producing bacteria (*Lactobacillus* species) and a thickened vaginal stratified epithelium (squamous epithelium) and mucus layer (protective mucus layer) are both a result of increased estrogen and glycogen levels in the body. Estrogen and glycogen levels in the body encourage the growth of lactic acid-producing bacteria, thicken the vaginal stratified epithelium, and also grow the protective mucus layer of the vagina [3]. *Lactobacillus*, specifically *Lactobacillus crispatus*, *Lactobacillus jensenii*, *Lactobacillus gasseri*, and *Lactobacillus iners*, dominate the vaginal microflora [4]. *Lactobacillus* acts as an antimicrobial agent against pathogenic microbes acts by creating an acidic environment in the vagina by producing lactic acid and acetic acid–thus creating a chemical barrier to various infections [5]. The presence of a high percentage of these bacteria in the vagina often corresponds to a healthy condition, whereas a low percentage or even absence corresponds to an abnormal or diseased condition. The microbiota is very important in preventing colonization of anaerobic and micro aerophilic pathogens in the vagina, depletion of lactobacilli or unbalancing of the vaginal microbiome, which cause bacterial vaginosis (BV). In non-pregnant women, the resident vaginal microbial flora has been found to be changed in a variety of disorders such as bacterial vaginosis (BV), related to an increased threat of upper genital tract and sexually transmitted infections and infection with HIV [6,7,8]. In pregnant women, BV enhances the risk of early miscarriage [9,10], late miscarriage [11,12], post-abortal sepsis [13], recurrent abortion [14], preterm prelabor rupture of membranes (PPROM) [15], histological chorioamnionitis [16,17], spontaneous preterm labor (SPTL) and preterm birth [18,19,20], and postpartum endometritis [21,22]. These studies, taken together, provide strong proof that microbiota in the vaginal tract are responsible for the variations in symptoms of the lower genital tract and incite a need for further investigation. In order to treat various diseases, modulation of microbial diversity by the investigation of prebiotics, probiotics or microbiota transplants as a therapeutic strategy is important [23]. Similarly, mapping the vaginal microbial communities in healthy females and recognizing changes in microbial configuration during vaginal disorders could revolutionize the way we treat vaginal disorders [24,25]. Furthermore, understanding the normal vaginal microbiota may alter our treatment approach, allowing us to place a greater emphasis on rebuilding a flexibly strong microbiome to reduce the host’s inclination to bacteria rather than destroying pathogenic bacteria with antibiotics, which results in the impairment of healthy microbiota [26].The investigation of vaginal microbiota is a fast-growing discipline, i.e., in investigations of the optimal techniques for identification of microbial communities in the lower genital tract. Approaches to microbiome research are increasingly diverse. Therefore, in this review we represent a summary of the numerous explorations that have pinpointed microbial communities present in the female lower genital tract. DNA-based approaches to understanding provide useful insights, especially when combined with community surveys and metagenomic data [27,28,29].

Studies based on samples collected from the posterior vaginal fornix, ecto- and endocervix regions to capture the vaginal microbiota have picked out a number of vaginal microbial communities that cannot be recognized by long-established conventional methods but can be revealed using 16S ribosomal RNA (rRNA) gene sequencing [30,31] and enlarged culture techniques [32,33,34]. In this review, we primarily focus on a widespread outline of the strategy that is presently used to study the vaginal microbiota and their combined genomes and talk about the components of experimental blueprint and data analysis. Scientists studying the vaginal microbiota will be able to benefit from this data. In general, we talk about sample collection considerations, laboratory procedures for recognizing microbiota, the prevention of microbial contamination, and bioinformatics methods for data processing and analyses. This review may provide an insight into approaches for evaluating the vaginal microbiota, making it simpler for researchers in the field to investigate, choose, and use procedures as needed The strategy adopted in this review added in supplementary Figure S1.

## 2. Considerations for Sample Collection

Microorganisms isolated from vaginal samples are assumed to reflect the microbiome found in the female vagina and vulva, as well as the area around the external genitalia. While vaginal samples are simple to obtain, important considerations must be made to expedite the acquisition of comprehensive microbiological information from these samples and to minimize sample contamination. Additionally, attempts should be made to collect samples throughout a study in a systematized manner to reduce unplanned variability. After collection, samples should be carefully stored and transferred in aseptic conditions. In fact, when comparing the make-up of a microbial community in a sample to that of its natural human habitat, the sample processing setup has a tremendous impact. For example, exposure to oxygen can stimulate cell death in strict anaerobes but can induce the propagation of aerobes within minutes [35]. Thus, throughout sample collection and processing, variables like oxygen tension, pH, and temperature should be controlled and standardized. After sample collection, specimens should be handled in a biosafety cabinet to minimize the risk of contamination.

### 2.1. Sample Collection Methods and Storage

Scientists should balance the benefits and downsides of various sampling procedures when planning an experiment to determine which approach is ideal for answering the scientific topic at hand. The procedure for obtaining vaginal specimens includes direct collection from the posterior vaginal fornix, ecto-, and endocervix regions using sterile vaginal swabs [5]. To investigate the vaginal microbiota, wipe the vaginal mucous, clean the cervix, and collect the secretions using a sterile swab. Samples were taken from the vagina using a culture swab under direct observation during speculum examination. Place the sample collection swab in vagina and gently rotate it clockwise for 10 to 30 s. Ensure that the swab touches the vaginal walls so that mucus is absorbed by the swab, and carefully withdraw the swab, avoiding touching the skin. Remove the cap from the sample collection swab and place it into the transport tube right away. Tightly, re-cap the swab specimen transport tube to minimize the risk of contamination and transport it to the laboratory at room temperature [36]. Immediately after collection, samples for sequencing should be aliquoted and kept ice cold to minimize undetectable batch effects that may occur during sample handling and to allow for the successive parallel processing of multiple samples. Sample storage temperatures of –20 °C and –80 °C had no significant effect on the composition of microbes in the vaginal specimens [37]. If instant freezing and storage are not an option, variations in sample composition can be introduced by freezing delay.

### 2.2. Sample Metadata

The human vaginal microbiome is constantly changing and is significantly influenced by the host’s surroundings. In fact, ageing [38], hormones [39], lifestyle [40], nutrition [41], alcohol consumption, smoking [42] and the use of contraception devices [43] have been found to affect the microbial composition of the vaginal cavity. Additionally, the menstrual cycle and sexual activity have been related to alterations in the composition of the vaginal microbiome [44].

After sample collection, several supportive techniques can be used to analyze the microbial constitution of a specimen, including conventional culture-dependent and culture-independent or metagenomics-based methods.

#### 2.2.1. Culture-Dependent Characterization of the Vaginal Microbiome

**Figure 1:**
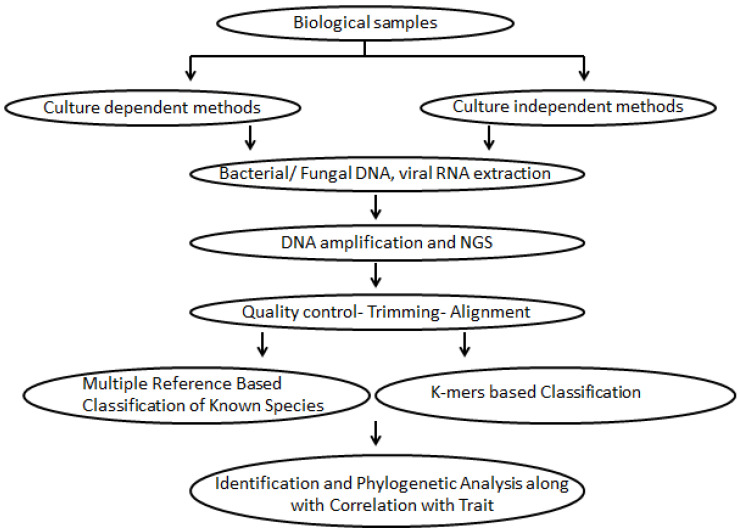
Workflow of High-Throughput Sequencing Analyses from Sample Collection to Correlation with Traits and Phylogenetics.

Traditionally, to investigate bacterial or fungal diversity, culture-dependent methods were used as shown in Figure 1. To determine the bacterial composition of the vaginal flora, collected swabs were used to form a vaginal smear, which was then heat-fixed and Gram-stained before being examined under the microscope. Some standardized criteria based on Nugent scoring and Amsel’s criteria were used to analyze the presence of Gram-positive *Lactobacillus,* which are more beneficial compared to Gram-negative organisms and other Gram-variable actinobacteria such as *Gardnerella*, *Atopobium* and *Mobiluncus* [45,46,47]. The Amsel test is based on the evaluation of four clinical conditions: the presence of white vaginal discharge, vaginal pH > 4.5, the whiff test, i.e., the production of fishy odour on the addition of 10% KOH to the sample, and the appearance of clue cells [47]. The Nugent score, on the other hand, is calculated by estimating large Gram-positive rods (*Lactobacillus*), small Gram-variable rods (*Gardnerella*), and curved Gram-variable rods (*Mobiluncus*), using the standardized method described by Nugent et al. and assigning a score of 0–10 [48]. For Nugent’s scores, the bacterias’ cell shapes and sizes are taken into consideration. A score of 0–3 is considered normal, with the predominance of *Lactobacilli* indicating healthy vaginal health; 4–6 is scored as an intermediate level of mixed Gram-negative/variable flora; 7–10 refers to the presence of no or little *Lactobacilli* and the predominance of Gram-negative/variable rods or curved rods; whereas a score of >7 indicates BV infection [46]. Compared to Amsel’s criteria, Nugent scoring has a higher sensitivity and is more reproducible when forecasting BV, but reading the slides requires a skilled laboratory and manpower [3].

Prior to the emergence of high-throughput metagenomics or culture-independent methods, these culture-based methods were utilized to explore the microbial composition of the vagina. Culture-based methods have various drawbacks: suitable culture conditions are not known, which leads to many microbial species being undetected; if microbial species have been isolated, they are unable to recognize and culture-based procedures are very monotonous and not adjustable to high-throughput investigation. Advances in DNA/RNA and protein analysis platforms have been utilized to uncover the real diversity of the vaginal microbiome, which had previously been underestimated due to the fact that less than 1% of bacteria survive and form colonies on agar plates [49]. Because most microbes are difficult to propagate in the laboratory using traditional culture-based methods, high-throughput molecular methods have played an important role in describing microbiota [50]. With the emergence of metagenomics-based techniques, identifying markers such as DNA are used for the identification of various microbial communities. Therefore, metagenomics-based methods have become the method of choice for investigating the human vaginal microbiota.

#### 2.2.2. Culture-Independent Methods

Many of our current understandings of the vaginal microbial flora are based on qualitative and semi-quantitative descriptive research using cultivation-dependent methodologies. The development and introduction of cultivation-independent molecular-based techniques have provided new information about the composition of normal vaginal flora, as well as abnormal colonization of the vagina in recent years—which has supplemented existing knowledge from cultivation-dependent techniques. Metagenomics-based techniques, also known as culture-independent procedures, are used to quickly and accurately describe microbial communities as a result of various microbial study methodologies, as shown in Figure 1. Using culture-independent methods for the identification of microbiota based on genomic DNA analysis, the DNA is isolated from specimens using a variety of next generation sequencing (NGS)-related tools. However, NGS was developed for broad spotting of microbial communities. These procedures can also be used to isolate individual microbial species from mixed cultures. After the isolation of DNA, two diverse methodologies were used to explore the microbiota. Whole-genome shotgun metagenomic analysis focuses entirely on the sequencing of microbial genomic DNA present in a designated sample; whereas marker gene sequencing targets sequencing of a particular locus in all genomes (the microbial gene region). The 16S rRNA gene, which is the most commonly used marker gene, is specific to archaea and bacteria, whereas the 18S rRNA gene and the 28S rRNA gene are specific for eukaryotic microorganisms, and for fungi, the internal transcribed spacer is used (ITS) [51]. This review focuses on various methods that are currently extensively approved for examining diverse microbial communities. Microbial DNA extraction, PCR amplification of the 16S rRNA gene, and NGS are among the techniques used.

#### 2.2.3. DNA Extraction of the Vaginal Microbiome

The extraction of DNA from microorganisms includes some basic steps, including cell lysis, elimination of non-DNA macromolecules, and DNA purification [52]. At the outset, lysis of the cell membrane is performed by using a combination of physical, chemical (using surfactants, detergents, and heat) and mechanical means (bead beating) [4,53,54] which accelerates the process of cell lysis in complex microbial groups [55]. Additionally, RNA and proteins can be broken down by using enzymes, i.e., RNases and proteases, to enhance DNA yield and restrict impurities from non-DNA cellular constituents [56,57]. Secondly, DNA is purified from various contaminants, including salts, cellular debris, proteins, and other existing cellular contaminants. The various techniques for DNA purification include isopropanol/ethanol precipitation or phenol–chloroform extraction, separation of aqueous-phase DNA from organic phase phenol-denatured proteins or solid phase DNA adsorption (silica) mini-column, which includes the modification of DNA binding by altering salt and pH levels [58]. The chosen DNA extraction procedure depends upon the microbial composition of the original sample. Due to the thickened cell walls of gram-positive bacteria and mycobacteria, it becomes harder to lyse the cells than in other microbial communities [59]. However, if too harsh a DNA extraction method is used (bead beating or sonication), the DNA extracted from the varied microbial species gets sheared [59]. Future studies differentiating discrete DNA extraction procedures to pick out the ideal method for microbial DNA isolation from vaginal samples have been accomplished for diverse samples that contain low and high volume microbial biomass such as urine samples [60], feces [61], and marine biofilms [62]. Prior to DNA separation, microbes inside a vaginal sample are condensed using centrifugation and filtration techniques, comparable to other samples of high and low microbial biomass, such as sea water or glacial ice [63] and air particles [64]. After concentration, various (such as Qiagen) DNeasy Blood and Tissue kits and MoBio Power Soil kits have been commonly used for vaginal microbiome studies [63,64,65,66].

#### 2.2.4. Choice of Universal PCR Primers

The selection of the primers and the barcode is critical in metabarcoding studies. So far, no ideal solution for capturing all microbes and all conditions has been accepted. There is a vast selection of PCR primers, each of which has its own benefits and drawbacks. The choice of PCR primers should be carefully taken into consideration, including the degree of phylogenetic information generated by the fragment, the desired taxonomic coverage, and the affinity of the fragment length with the sequencing method, as well as the amount of selectivity for amplifying microbial sequences in contrast to host sequences. Universal marker genes for community profiling of bacteria, such as the 16S rRNA gene, enhances the efficiency of sequencing [67,68]; marker genes for eukaryotic microorganisms include the18S rRNA gene and the 28S rRNA gene, whereas the usual target for fungi is the nuclear ribosomal internal transcribed spacer ITS [51,69]. The two internal transcribed spacers, including ITS1 and ITS2, flanking the 5.8S ribosomal subunit, both give features for the identification of fungi. ITS1 and ITS2 differ in the validity of strain prediction. ITS1-based PCR favors Basidiomycota, whereas ITS2-based PCR has a taxonomic bias towards Ascomycota [70]. In fungi, the ITS regions vary in length between 200 bp and 800 bp, which impacts the PCR potency and influences the sequencing technologies [71,72]. Ongoing research is being carried out to improve the resolution of PCR primers. The correct choice of primer would enhance the taxonomic resolution power and enhance the validity of species recognition. Another critical consideration is the choice of a hypervariable region in the 16S rRNA gene that is to be amplified by the PCR primers [73,74].

Since no single hypervariable region can distinguish all bacterial strains, different hypervariable regions need to be targeted during analysis [74]. Therefore, to understand and analyze the vaginal microbiota, various hypervariable regions have been used, including V1/V2(27 F/338R) [75], V3/V4(319 F/806R), V1–V3 [76,77], and V3-V5(341F/926R) [78]. Due to the over-representation or under-representation of hypervariable regions in the defined microbial taxa, the correct selection of these is important for taxonomic identification [79,80]. The targeting of different hypervariable regions by primers has yet to be performed for the vaginal microbiota. In comparison to the V1/V2 region, which is used to identify *Lactobacillus jensenii*, *Pseudomonas gessardii*, and *Megasphaera elsdenii* but is ineffective against, e.g., *Gardnerella vaginalis*, *Bifidobacterium bifidum*, and *Chlamydia trachomatis* [81]; the V3/V4 hypervariable region primers do not have this constraint [82].

## 3. Sequencing Methodologies

New high-throughput NGS methods have made it possible to study microbiota at unprecedented scales. NGS of the 16S rRNA gene is used to recognize the DNA sequences of various types of microbiota in a sample. Our capacity to investigate microbiota has been changed by the introduction of high-throughput NGS technologies. NGS is used to identify the DNA sequences of distinct types of microbiota in a specimen by sequencing the 16S rRNA gene. [83,84]. The 16S rRNA gene is an excellent target for studying bacterial diversity because it has nine hypervariable areas that can be utilized to distinguish species based on individual nucleotide variations. NGS’s use of universal primers to conserved areas adjacent to each other suggests both large (phylum level) and fine (genus, species, and strain) distinctions. [82]. Although the 16S-based method sequences a few hypervariable regions at a time, as a result, they are restricted to a shorter read length in contrast with Sanger sequencing. The various approaches and their approval by the Human Microbiome Project (HMP) Consortium identified that, based on the sequencing of different hypervariable regions, there is an alteration in the taxonomic profile. For example, the V3–V5 amplicon reveals both Acinetobacter and Escherichia genera, but V1–V3 fails to express these genera [85]. Furthermore, while V6V9 may underrate Bacteroides, it provides adequate coverage for *Escherichia coli* and *Pseudomonas*. In contrast to vaginal microbiome data from the HMP, which reveals that V1–V3 expresses communities of predominant *Lactobacillus* species, whereas V3–V5 amplicons reveal either lactobacilli-diminished or lactobacilli- dominant groups [86]. In comparison to V3–V5, V1–V3 does not fully differentiate between the Enterobacteriaceae family and the Staphylococcus genera [87]. However, a number of studies have used V6, V7, V8, and V9 regions to indicate good coverage of microbial communities as compared to vaginal microbiota. Metagenomics is a broad term that refers to the field as a whole, as well as the specific sequencing of whole-community DNA. It is naturally supplemented by metatranscriptomics (cDNA sequencing) and functional technologies such as metaproteomics and community metabolomics. Researchers can investigate the actively transcribed ribosomal and messenger RNA from a community using metatranscriptomics. On the other hand, metabolomics deals with the analysis of metabolites produced by one or more organisms in a particular environmental and physiological condition by characterizing them via mass spectroscopy, NMR, or other analytical methods [88]. Current host–microbe–microbiome systems rely on various models of commensal microbes, pathogens, and hosts for interaction modelling, which is especially important for human health. Some of the studies focusing on the vaginal microbiome with different particulars are summarized in Table 1.

Current sequencing technologies include 454 Roche (pyrosequencing), capillary Sanger sequencing, and Mi Seq or Hi Seq (Illumina)–each with unique characteristics such as read length, insert size, sequence accuracy, usability, speed, and cost [104,105]. Technological advancements include new sequencing platforms such as Pac Bio (Pacific Biosciences), Ion Torrent (Thermo Fisher Scientific), and MinION (Oxford Nanopore Technologies) sequencers. The precision of sequence-based microbial profiling depends on the characteristics of the sequencing data. The NGS technologies that originate sequencing errors lead to incorrect base calls in the final sequences that make it difficult to differentiate between biological variability and technical variability [106,107], which results in incorrect taxonomic classification [108] and inflated estimates of microbial diversity [109]. The correction of sequencing errors is a unique challenge in 16S rRNA gene sequencing output [110,111] that has evoked the evolution of bioinformatic tools, particularly for 16S rRNA gene sequencing output. The approaches to correcting the sequencing errors depend on the use of specific sequencing technologies. The development of various 16S rRNA gene sequence correction tools, used with pyrosequencing technology, is characterized by deletions, insertions, and homopolymers [112,113] and are inappropriate for Illumina sequencing data [114,115]. Two methods for correcting Illumina sequencing errors are merging overlapping paired-end reads and quality trimming [111]. Typically, 16SNGS sequencing is performed on one of the gene’s variable regions. Consequently, short sequencing reads (typically spanning around 300–500 bases) via 16SNGS would not be appropriate for species determination of some bacterial genera. Despite the fact that short reads from NGS platforms are more precise, research comparing the output of NGS and long-read sequencing techniques have revealed that the latter produces more taxonomic categorization at the genus and species levels. Furthermore, even in the variable parts of their 16S rRNA sequences, certain bacteria may show a high degree of similarity to other members of the same family. Sequencing for additional genes will lead to a more accurate identification of species for these bacteria [116].

It has been demonstrated that the primer used for the 16S RNA sequencing has a significant effect on vaginal microbiota profiling. Moreover, the sequencing methods reveal the existence of *Lactobacilli* only at the genus level. Due to low sequence variation, studies utilizing particular sections of the 16S rRNA gene are unable to distinguish lactobacilli beyond higher level taxonomic categorization. Additionally, the findings indicate that identification of *L. iners* using 16S rRNA amplicon sequencing may overestimate the existence of this species. The 16S rRNA gene has impeded the identification of microbial communities due to intrinsic variations in community profiles created by sequencing of various hypervariable areas, short read lengths, and taxonomic categorization issues due to low resolution for closely related species [117]. The use of whole genome sequencing of vaginal samples enables the exploration of “gene-centric” and “taxonomy-centric” profiles of microorganisms’ metabolic and pathogenic capabilities, as well as the characterization of numerous bacterial taxa in selected vaginal samples. It is beneficial to obtain knowledge about the genetic makeup of fastidious organisms that have not yet been cultivated [118,119]. This technique has the potential to become a critical weapon in the fight against antibiotic resistance, a serious challenge to modern healthcare [120]. However, the approach has several drawbacks. It is significantly more expensive to acquire comparable profiles of a microbial community using whole genome metagenomic sequencing than with amplicon sequencing, especially when a large proportion of the metagenomic sequencing effort is ‘wasted’ on the frequently >90% human DNA present in vaginal microbiome samples [121]. Despite the fact that the full genome has been sequenced, it cannot be assembled entirely if the genome contains repeating lengths of DNA that exceed the length of DNA that can be sequenced in a single read using the sequencing method used [119,120]. Some of the studies focusing on the vaginal microbiome with different particulars are summarized in Table 1.

## 4. Bioinformatics Data Analysis Tools

The DNA sequences must be interpreted and examined after the sequence is obtained. Modern bioinformatics tools are required for the processing and analysis of large volumes of sequence data; direct data manipulation, such as manually aligning DNA sequences, is no longer possible. Many methods for analyzing sequence data are available, including amplicon sequence variant detection, OTU clustering, chimaera eradication, and taxonomy assignment. We distinguish between the examination of targeted amplicon and metagenomic vaginal microbiome data in this review paper.

### 4.1. Pre-Processing and Signal Extraction

To begin with, pre-processing the sequencing data is necessary to provide the outline required for further processing stages. In this step, if the samples are multiplexed—which includes multiple microbiome specimens being combined on a single sequencing run—first de-multiplexing of the sequence reads can be performed [122] and then elimination of the primer sequences. Pre-processing also includes sequence quality filtering to avoid bias and reduce artefacts caused by sequencing and PCR amplification [123]. Low-quality reads, such as anticipated errors, unclear bases, mismatched bases in barcodes and primers, and sequence length, can be avoided using quality scores [124]. Prior to quality filtering, when sequencing creates overlapping paired-end reads, they can be combined to produce improved posterior quality scores. This also decreases the error rate by eliminating sequences with low abundance and poor coverage.

### 4.2. Analysis Tools for Targeted Amplicon Data

For the analysis of 16SrDNA sequence data, tools including Quantitative Insights Into Microbial Ecology QIIME [125], Mothur [123,126] and Genboree [85] are frequently used by researchers to analyze and compare vaginal microbiomes using large quantities of DNA sequence data. Furthermore, various bioinformatics software for 16S rRNA gene sequencing data exists [127,128]. This programme allows users to create workflows without having a thorough understanding of the complexities involved in each phase of data processing and analysis. Well-documented QIIME and Mothur, both of which are supported by online communities, are excellent resources for learning the required procedures for resolving technological issues and data processing. The bioinformatics workflow is documented in full (including the version, technique, and parameters used for each tool), which is important for maintaining accuracy in bioinformatics processing and to promote repeatability and analytical transparency in vaginal microbiome research. To support the sharing of data analysis and documentation, numerous options exist. QIIME users should generate Jupyter Notebooks and Mothur users can create a batch file to document the workflow. As previously stated, high-throughput sequencing technologies result in sequence data mistakes. The sequencing findings for 454 pyrosequencing technology were created by grouping the flow grams into a smaller number of DNA sequences that were presumably present in the native biological samples. Tools such as AmpliconNoise [129] and Denoiser [130] can be applied to reduce these errors individually. Furthermore, in the statistical programming package R, if downstream analyses are accomplished, R Markdown can be used to authorize the sharing of key outputs and underlying code for analyses.

### 4.3. Operational Taxonomic Units (OTU) Grouping

To decrease the degree of downstream calculations and noise, DNA sequence data can be clustered into OTU. OTU clustering methods are mostly used for 16S rRNA gene sequencing, based on their similarity, DNA sequencing can be grouped into OTUs for the estimation of diversity. The choice of a clustering method and similarity threshold has a significant impact on the downstream results, with a 97% similarity threshold being the most commonly used [131]. De novo, closed reference and open reference are the three categories of OTU clustering algorithms [132]. De novo methods perform grouping based on the similarity of sequences in the data set to one another [131,133]. Closed-reference approaches group sequences based on similarity to a reference database [132], and thus, the results will depend on the used database. As few of the bacteria found in vaginal samples are included in existing reference databases, this approach is difficult to apply in microbial profiling of these specimens. Open-reference approaches eventually achieve closed-reference OTU clustering before doing de novo OTU clustering on sequences that do not group using a reference database [134]. All of the published vaginal microbiome research used OTU clustering to estimate the number of bacteria present in a sample, with the majority of the studies detecting thousands of unique OTUs from mock communities with only 100 actual OTUs [133,135]. 

Amplicon sequence variant (ASV) methods, which attempt to achieve greater taxonomic resolution than OTU clustering methodologies, have been developed as an alternate strategy for OTU clustering [135]. These methods look at the frequency distribution of sequences in order to find and fix Illumina sequencing errors. Several ASV algorithms like Deblur [136] and UNOISE2 [137] utilize previous models of sequencer error profiles, whereas MED [138] utilizes information theory to differentiate closely related taxa and DADA2 [139] utilizes experiment-specific adaptive error models that are evaluated from the data. The sequence dissimilarity can be investigated to see if it is biologically significant once the sequencing errors have been verified and corrected [135]. Furthermore, ASV methods outperform OTU clustering approaches in terms of estimating microbial diversity [135,139]. Moreover, ASV approaches are more consistent and long-lasting across research, and they are not reliant on reference databases [107,136,137]. Due to their high resolution, they may be especially effective for vaginal microbiome studies, as they can distinguish real bacterial DNA sequences from contaminants to a larger extent than the OTU clustering method. Table 2 listed some of the in-silico tools that are necessary for data analysis.:

## 5. Conclusions

The vaginal microbiota appears to play a key role in vaginal physiology and pathogenesis, implying that its impact on the host immune system should be investigated further. This conceptual revolution, on the other hand, will require a thorough understanding of the microbial population that lives in the female genital tract. To do this, significant efforts must be made to identify new taxa through cultures in order to uncover the remaining 80% of undiscovered microbiota that are regarded un-culturable. The development of validated technology for studying the microbiota, including nucleotide extraction techniques and better database quality, is critical to achieving this goal and must be addressed. Furthermore, this microbiome must be included in databases routinely utilized in metagenomic research. The vaginal microbiota seems to be altered between women with and without vaginal infection. Furthermore, the vaginal microbiota of women with bacterial vaginosis (BV) was shown to be different from that of women who did not have BV. It also seems to have changed in proportion to the proportion of females suffering from sexually transmitted infections, preterm birth (PTB), early miscarriage, post-abortal sepsis, and postpartum endometriosis. These investigations show that the vaginal microbiome is likely clinically important and should be investigated further. The technical and computational complexities of assessing low microbial biomass conditions must be examined, as the involvement of the vaginal microbiota in various illnesses, as well as the potential therapeutic prospects it brings, continues to be clarified. To obtain reliable and repeatable conclusions from studies to better understand these microbial populations and their links with vaginal diseases, thorough sample collection and data processing will be required. This review provides an initial framework to aid in the development of community guidelines and underlines the possibilities for female health in this growing and highly interdisciplinary field of study.

## Figures and Tables

**Table 1:** Metagenomics studies with different collection sites in the vagina and sequencing techniques adopted, with observations from the data generated.

Serial No.	Study	Study Aim	Sample No.	Sample Site	Technique	Primer Used	Analysis Software/Tools Used	Findings
1	[37]	Investigation of the effect of storage conditions on the vaginal microbiota	N = 8	Mid-vagina	454 Life Sciences FLX sequencing(Pyrosequencing)	V1–V2 27F 338R	QIIME software UCLUST software	At ultra-low temperatures (−80 °C) or storage for one week at (−20 °C) prior to storage at (−80 °C) for four weeks, no significant changes were observed when compared to non-frozen samples.
2	[89]	Characterization of the vaginal microbiota of women with preterm labor (PTL) and preterm pre-labor rupture of membranes (PPROM)	N = 65	Posterior fornix	Illumina 16S rRNA gene sequencing	V3–V4 319 F 806 R+	QIIME software package (v. 1.8) SPSS software ver. 21.0	The microbial abundance and diversity in the PPROM was higher than in PTL women.
3	[90]	To explore the profiling of the vaginal microbiota associated with HPV16 infection (control)	N = 52 27 HPV16 + ve and 25 HPV − ve	Vaginal fornix and cervix	Illumina Hiseq X-ten platform shotgun metagenomic sequencing		SOAPdenovo (Version 1.05) Software MetaGeneMark	The abundance of *Lactobacillus* (Firmicutes) was lower in HPV16-positive women than in controls.
4	[91]	Identification of vaginal microbial signatures in women with PTL	N = 1572	Vaginal and rectal samples	Illumina MiSeq	V1–V3	ASGARD, HUMAnN2 and ShortBRED	Coupled with genetic factors, microbiome-associated taxonomic, metabolic and immunologic biomarkers may be useful in defining the risk of PTL.
5	[92]	Vaginal microbial gene catalogue differs in pregnancy	N = 68	Vaginal introitus, posterior fornix, and mid- vagina	454FLX Titanium platform	V3–V5 354F 926R	QIIME	Characterization of healthy, gravid vaginal microbiome.
6	[93]	Characterization of changes in the composition of the vaginal microbiota of pregnant women	N = 54	Posterior fornix	Pyrosequencing	V1–V2 27F 338R	UCHIME UCLUST	Vaginal microbiota in normal pregnancy is different and stable from that of non-pregnant women.
7	[94]	To examine composition of the vaginal microbiome of women of African and non-African ancestry differently during pregnancy	N = 2582	Vagina (Self-sampling)	Roche 454 titanium Illumina MiSeq	V1–V3	CLARK-S MetaPhlAn2	Women of European, African and Hispanic ancestry exhibit different vaginal microbiome compositions and dynamics during pregnancy
8	[95]	To determine the vaginal bacterial composition in healthy Nigerian women and BV women	N = 28	Vagina	16S rRNA sequencing	V4 region	QIIME-UCLUST	Characterization of vaginal bacteriome compositions of healthy and often times annoying condition known as BV in Nigerian women.
8	[4]	Understanding of the composition and ecology of the vagina microbial ecosystem in asymptomatic women	N = 396	Vagina	454 Life Sciences FLX sequencing	V1–V2 27F 338R		Differences in vaginal bacterial community composition in different ethnic group of North American women.
9	[96]	Comparison of vaginal microbiomes of African American women with women of European ancestry with and without a diagnosis of BV	N = 1268 AAW N = 416 EA	Mid-vagina	Roche 454 GS FLX Titanium	V1–V3		African women have high rates of BV.
10	[97]	To examine the composition of the vaginal microbiome throughout pregnancy and in the postpartum period	N = 42	Posterior fornix	MiSeq sequencing	V1–V2 28F 388R	Mothur	Biogeographical and ethnic differences exist between microbial communities in the vaginal microbiome during pregnancy and in the postpartum period.
11	[98]	Pregnant women at high and low risk of PTB were studied.	N = 88	Posterior fornix	Sanger sequencing	8F 1492R	QIIME	PTB is related to the variety of the vaginal microbiome during human pregnancy, and race/ethnicity and sampling site are relevant determinants.
12	[99]	To assess the vaginal microbiome throughout full-term uncomplicated pregnancy	N = 12	Posterior fornix and cervix	Illumina MiSeq	V3–V5 357F 926R	IM-TORNADO QIIME	Normal pregnancy has a less diverse and highly stable microbiome.
13	[100]	Characterization of the vaginal microbial community in African-American, pregnant women associated with the risk for preterm birth.	N = 149	Mid-vagina	Roche 454	V1–V3 27F 534R V3–V5 357F 926R		Preterm birth is linked to decreases in the richness and variety of the vaginal microbial community in the African-American population.
14	[101]	Comparison of changes in the vaginal microbiota and metabolome of females as a result of frequent genital illnesses.	N = 79	Vagina	NGS	V3–V4	PANDAseq (v. 2.5.0) QIIME pipeline (release 1.8.0)	Women with vulvovaginal candidiasis (VVC) and Chlamydia trachomatis infection (CT) had a vaginal microbiome that was positioned between eubiosis (healthy women) and dysbiosis (BV-positive subjects), with *lactobacilli* depletion and an increase in several anaerobe genera (e.g., *Gardnerella*, *Megasphaera*, *Roseburia*, and *Atopobium*
15	[102]	To study the relationship between the vaginal microbiota and CIN disease progression	N = 169	Posterior vaginal fornix	Illumina MiSeq sequencing	V1–V2	Mothur	Vaginal microbial diversity is associated not only with HPV infection, but also with advancing cervical intra-epithelial neoplasia (CIN) severity
16	[103]	To discover the vaginal microbiome of postmenopausal women who were either healthy or experienced vaginal dryness	N = 500	Mid-vagina	Illumina sequencing	V6	Uclust version 3.0.617	In women with moderate to severe vaginal dryness, there is an inverse relationship between *Lactobacillus* ratio and dryness, as well as an increase in bacterial diversity.

**Table 2:** Widely used in-silico tools for the microbiome Analyses.

Serial No.	In-Silico Tools	Functions	URL	References
1	QIIME	Used to perform demultiplexing, quality filtering, operational taxonomic unit picking, taxonomic assignment, phylogenetic reconstruction, diversity analyses and visualizations	http://qiime.org/	[125]
2	Mothur	Used to analyze raw sequences to the generation of visualization tools to describe α and β diversity	http://mothur.org/	[126]
3	VAMPS	VAMPS is the collection of tools used to visualize and analyze data for microbial population structures and distributions	https://vamps2.mbl.edu/	[140]
4	FastTree	Command line tool used to generate phylogenetic trees by maximum-likelihood from nucleotide and protein sequences	http://www.microbesonline.org/fasttree/	[141]
5	BLAST	Tool used to find similarity between nucleotide or protein sequences with reference database	https://blast.ncbi.nlm.nih.gov/Blast.cgi	[142]
6	MG-RAST	Open source application used for the phylogenetic and functional analysis of metagenomic data	https://www.mg-rast.org/	[143]
7	IMG/M	Analysis and annotation of genome and metagenome datasets	https://img.jgi.doe.gov/cgi-bin/m/main.cgi	[144]
8	iMAP	This is a bioinformatic pipeline that performs metadata profiling, quality control of reads, sequence processing and classification, and diversity analysis of operational taxonomic units	https://github.com/tmbuza/iMAP	[145]
9	Phyloseq	Phyloseq is an R programming language package used to import, store, analyze, and graphically display complex phylogenetic sequencing data that has already been clustered into Operational Taxonomic Units (OTUs).	https://joey711.github.io/phyloseq/	[146]
10	SILVA	Database of ribosomal RNA database with web based tools used for sequence alignment and many interactive analysis	https://www.arb-silva.de/	[147]
11	HUMAN 3.0	Used for profiling the microbial metabolic pathways and other molecular based functions from metagenomic or metatranscriptomic data	https://huttenhower.sph.harvard.edu/humann	[148]
12	PICRUSt	This software is used to predict metagenome functional content from marker gene and full genomes.	http://picrust.github.io/picrust/	[149]
13	Meta Gene Mark	Tool is used to identify the protein coding regions from the metagenomic sequences.	http://exon.gatech.edu/Genemark/meta_gmhmmp.cgi	[150]
14	Glimmer-MG	Gene finding tool for microbial (bacteria, archaea and viruses) DNA	https://github.com/davek44/Glimmer-MG	[151]

## Supplementary Materials

The following are available online at https://www.mdpi.com/article/10.3390/life11111229/s1, Figure S1: workflow showing the methodology adopted for the evaluation of literature related with vaginal microbiome.
