# Supplementary material for: An Insight into Vaginal Microbiome Techniques
Source: Life (Basel). 2021 Nov 13;11(11):1229. doi: 10.3390/life11111229 (PMC8623751; doi:10.3390/life11111229)
Supplement: Supplementary file 1 [file life-11-01229-s001.zip › life-1396414-supplementary.pdf]

life

Supplementary material of An Insight towards Vaginal Microbiome Techniques

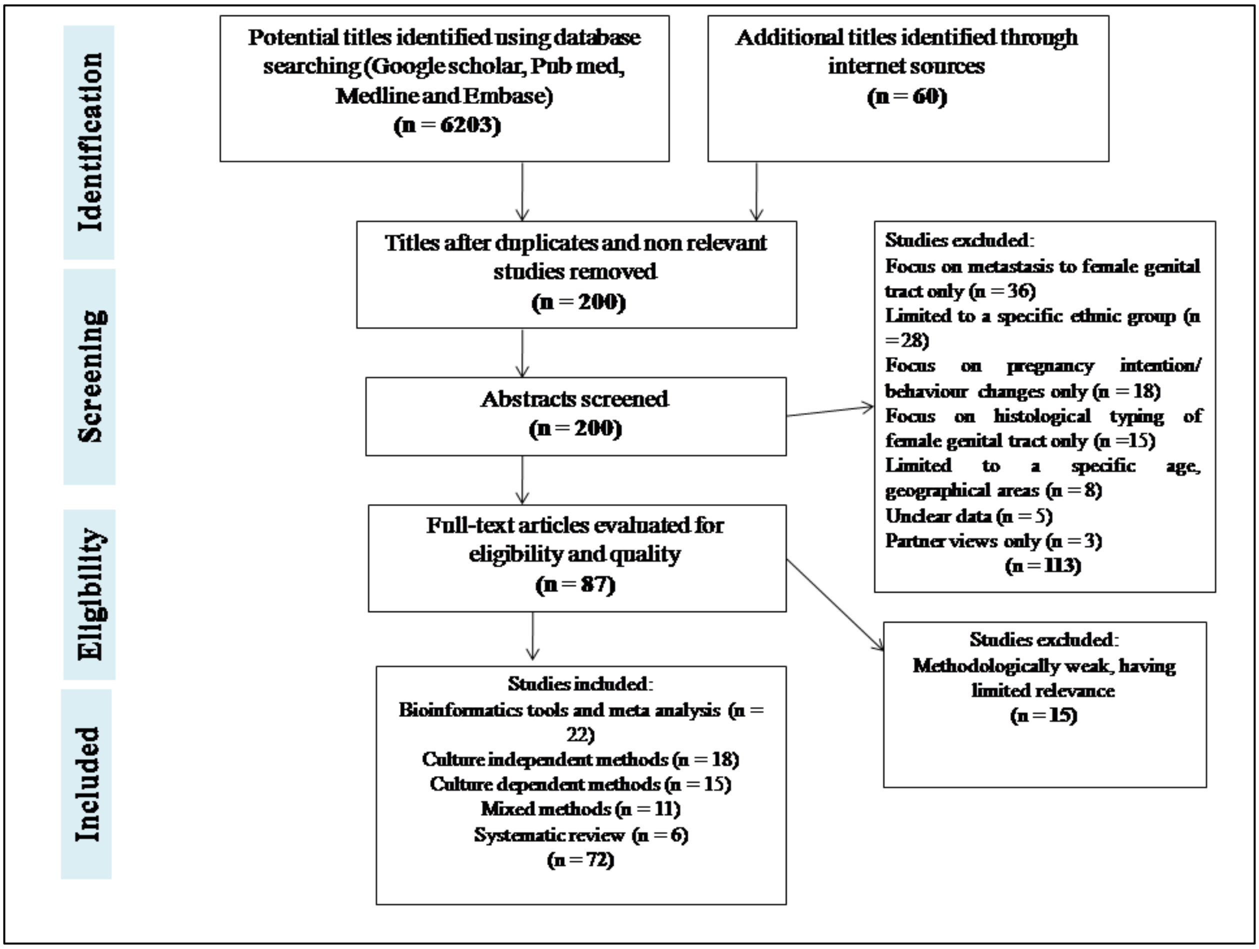

Figure S1. Workflow showing the methodology adopted for the evaluation of literature related with vaginal microbiome.

1. Materials and methods:

1.1. Search methods:

From November 2019 to July 2020, a literature review was carried out. The following search strategy was used to search databases such as Google scholar, PubMed, Medline, and Embase, Pubmed Central.

Microbiome/; Vaginal infection.ti,ab.; Vaginal health/; Microbial communities*; Vaginal microbiota.ti,ab.; Lactic acid producing bacteria/; Bacterial vaginosis*; Early miscarriage/; Preterm prelabor rupture of membranes/; Postpartum endometritis/; Preterm birth/; Microbial diversity/; Posterior vaginal fornix/; Bacterial flora/; *Lactobacillus*/; Healthy microbiota/; Conventional methods/; Vaginal cavity; Nugent scoring/; Amsel's criteria/; Female infertility

Keywords used to find vaginal microbiome-related metagenomics research:

Vaginal metagenomeic based/; 16S r RNA/; Bioinformatics methods/; Data processing/; Data analysis/analyses; Metagenomic/; Next Generation Sequencing/; 18S rRNA gene/; PCR amplification/; Microbial DNA extraction/; PCR Primers/; Phylogenetic information/; Community profiling/; Internal transcribed spacer/; Hypervariable region/HVR; V1/V2 , V3/V4 and V3-V5/; Operational Taxonomic Units/

1.2. Inclusion criteria

Studies that used culture-dependent and culture-independent methods to identify the vaginal microbiome, as well as bioinformatics and meta-analysis studies, were included, if they looked at microbiome alterations linked to normal and abnormal lower genital tract conditions.

1.3. Exclusion criteria

Studies that were not written in English were excluded. Focus on metastasis to the female genital tract only, limited to a specific ethnic group, focus on pregnancy intention/behavioral changes only, focus on histological typing of the female genital tract only, limited to a specified age, geographical locations, and so on were also omitted from the study. Data is ambiguous, and only the viewpoints of partners are available.
